# Prevalence and Patterns of Cosmetic Dermatological Procedures in Jazan, Saudi Arabia: A Cross-Sectional Study

**DOI:** 10.7759/cureus.71223

**Published:** 2024-10-10

**Authors:** Radwan Abu Taleb, Hasan Hannani, Mohammed E Mojiri, Osama A Mobarki, Sarah A Daghriri, Amani A Mosleh, Asail O Mongri, Jawaher S Farji, Alaa A Alzahrani, Abdulrahman M Safhi, Ola A Farhan

**Affiliations:** 1 Dermatology, Jazan General Hospital, Jazan, SAU; 2 Dermatology, King Fahad Central Hospital, Jazan, SAU; 3 College of Medicine, Jazan University, Jazan, SAU; 4 College of Medicine, Fakeeh College for Medical Sciences, Jeddah, SAU; 5 College of Medicine, Al-Rayan Colleges, Medina, SAU

**Keywords:** aesthetic procedures, attitudes, cosmetic dermatology, jazan, prevalence, public health, saudi arabia

## Abstract

Background: Cosmetic dermatological procedures are increasingly popular worldwide, reflecting changing societal norms and technological advancements. This study aims to investigate the prevalence and patterns of cosmetic procedures in Jazan, Saudi Arabia, and to explore related demographic, psychological, and attitudinal factors.

Methods: This cross-sectional study involved 439 participants from Jazan. Data were collected using a structured questionnaire covering demographic characteristics, psychological and dermatological conditions, experiences with cosmetic procedures, and attitudes towards them. Descriptive statistics were used for data analysis.

Results: Among the participants, 103 (23.5%) reported having undergone cosmetic dermatological procedures. The majority of these individuals were aged 18-30 years (394, 89.7%) and had attained a diploma or bachelor’s degree (237, 54.0%). Satisfaction with procedures varied, with 12 (2.7%) dissatisfied and 52 (11.8%) satisfied. Future intentions to undergo cosmetic procedures were expressed by 62 (14.1%) of participants. Attitudes towards cosmetic procedures included concerns about unrealistic beauty standards (221, 50.5%) and perceptions of affluence (256, 58.3%).

Conclusions: The study reveals a notable prevalence of cosmetic procedures in Jazan, with younger and more educated individuals showing higher engagement. While satisfaction levels are generally positive, diverse attitudes reflect ongoing debates about beauty standards and societal impacts. These findings highlight the need for continued research and tailored public health strategies to address the evolving landscape of cosmetic dermatology in Saudi Arabia.

## Introduction

Cosmetic dermatological procedures have seen a significant rise in popularity worldwide, reflecting growing societal interest in aesthetic enhancements and personal appearance. These procedures, which range from minimally invasive treatments such as chemical peels and Botox to more invasive surgeries like liposuction and rhinoplasty, have become increasingly accessible and socially accepted. The burgeoning demand for these services is driven by factors including advances in technology, increased media exposure, and evolving beauty standards [1-6].

In Saudi Arabia, a country with a rapidly modernizing society and an increasing emphasis on personal appearance, the prevalence of cosmetic dermatological procedures is of particular interest. The cultural and economic dynamics in Saudi Arabia may influence individuals' decisions to pursue such procedures, making it essential to understand the scope and nature of these practices within specific regions. Jazan, a city in the southwestern part of Saudi Arabia, presents a unique setting for examining the prevalence of cosmetic dermatological procedures. As a region with distinct socio-economic and cultural characteristics, Jazan offers insights into the local trends and attitudes towards cosmetic enhancements. Investigating these trends in Jazan can provide valuable data on the regional differences within Saudi Arabia and contribute to a more comprehensive understanding of cosmetic dermatological practices across the country.

Despite the increasing popularity of cosmetic procedures, there is limited research focusing on their prevalence and associated factors within Saudi Arabia, particularly in regions like Jazan. Understanding the demographic, psychological, and socio-economic factors influencing cosmetic procedure use is crucial for tailoring public health initiatives and providing relevant information to healthcare providers and policymakers [7-9].

This study aims to fill the gap in the literature by providing a detailed analysis of the prevalence and patterns of cosmetic dermatological procedures in Jazan. By examining demographic variables, psychological and dermatological conditions, experiences with cosmetic procedures, and attitudes towards these practices, the study seeks to offer a comprehensive overview of the current landscape of cosmetic dermatology in the region. The findings will contribute to the broader understanding of cosmetic procedure trends in Saudi Arabia and inform future research and healthcare strategies.

## Materials and methods

Study design and setting

This cross-sectional study was conducted in Jazan, Saudi Arabia, to investigate the prevalence of cosmetic dermatological procedures and related factors. Data collection occurred over a specified period, ensuring a representative sample of the population within the region.

Study participants

Participants were recruited using a non-probability sampling method. Inclusion criteria required participants to be residents of Jazan, aged 18 years or older, and able to provide informed consent. Individuals who were not residents of Jazan or were unable to consent were excluded from the study.

Data collection

Data were collected using a structured questionnaire, which included sections on demographic characteristics, psychological and dermatological conditions, cosmetic procedure experiences, and attitudes towards cosmetic procedures. The questionnaire was designed based on existing literature and expert consultation to ensure relevance and comprehensiveness (see Appendices).

Demographic characteristics included questions about age, gender, education level, work sector, family income, marital status, nationality, and city of residence. Psychological and dermatological conditions were assessed by asking participants about any previous psychological diagnoses, chronic diseases, and chronic skin conditions.

Cosmetic procedure experiences were evaluated by inquiring whether participants had undergone any cosmetic dermatological procedures, their level of satisfaction with these procedures, their future intentions regarding such procedures, and any complications experienced. Attitudes towards cosmetic procedures were assessed through questions about participants' perceptions of cosmetic procedures, including their views on beauty standards, vanity, psychological impacts, and signs of affluence.

Data analysis

Descriptive statistics were used to summarize demographic characteristics, psychological and dermatological conditions, experiences with cosmetic procedures, and attitudes towards cosmetic procedures. Frequencies and percentages were calculated for categorical variables. Data analysis was performed using Statistical Package for the Social Sciences (IBM SPSS Statistics for Windows, IBM Corp., Version 25.0, Armonk, NY), and results were presented in tables with appropriate summaries of the findings.

## Results

Demographic characteristics

The study population comprised 439 participants from Jazan, Saudi Arabia. Among the participants, 331 (75.4%) were female and 108 (24.6%) were male (Table 1). The majority were aged between 18 and 30 years (394, 89.7%), with the remaining participants divided into 31-45 years (34, 7.7%) and 46-59 years (11, 2.5%). In terms of education level, most participants had attained a diploma or bachelor’s degree (237, 54.0%), while 188 (42.8%) had secondary education. Only seven (1.6%) had completed a master’s degree or higher, with an equal number (7, 1.6%) having intermediate education or below (Table 1). The work sector distribution showed that 371 participants (84.5%) were unemployed, while 55 (12.5%) worked in the government or semi-government sector. Only eight (1.8%) worked in the private sector, and three (0.7%) were retired. Regarding marital status, 382 participants (87.0%) were single, 53 (12.1%) were married, one (0.2%) was widowed, and three (0.7%) were divorced (Table 1).

**Table 1:** Study population characteristics (N=439) Data are represented as frequencies and percentages for the demographic characteristics of the study participants.

Variable		Frequency	Percent
Gender	Female	331	75.4
	Male	108	24.6
Age Group	18-30 years	394	89.7
	31-45 years	34	7.7
	46-59 years	11	2.5
Education Level	Secondary	188	42.8
	Diploma/Bachelor	237	54.0
	Master's/PhD	7	1.6
	Intermediate and Below	7	1.6
Work Sector	Private Sector	8	1.8
	Government/Semi-Government	55	12.5
	Unemployed	371	84.5
	Retired	3	0.7
Family Income	5000-10000 SAR	102	23.2
	10001-15000 SAR	93	21.2
	15001-20000 SAR	57	13.0
	Less than 5000 SAR	137	31.2
	More than 20000 SAR	50	11.4
Marital Status	Single	382	87.0
	Married	53	12.1
	Widowed	1	0.2
	Divorced	3	0.7
Nationality	Saudi	434	98.9
	Non-Saudi	5	1.1

Psychological and dermatological conditions

In the sample, 405 participants (92.3%) reported no previous psychological diagnoses. Among those with psychological conditions, 14 (3.2%) had depression, 13 (3.0%) had anxiety, three (0.7%) had social phobia, and four (0.9%) had other conditions. Regarding chronic diseases, 396 participants (90.2%) reported no chronic illnesses, whereas 43 (9.8%) had one or more chronic diseases. For chronic skin conditions, 336 participants (76.5%) reported no such conditions. Eczema was reported by 26 participants (5.9%), acne by 46 (10.5%), and four (0.9%) reported other skin conditions (Table 2).

**Table 2:** Psychological and dermatological conditions (N=439) Data are represented as frequencies and percentages for psychological and dermatological conditions among the study participants.

Variable		Frequency	Percent
Previous Psychological Diagnosis	None	405	92.3
	Depression	14	3.2
	Anxiety	13	3.0
	Social Phobia	3	0.7
	Other	4	0.9
Chronic Diseases	No	396	90.2
	Yes	43	9.8
Chronic Skin Diseases	None	336	76.5
	Eczema	26	5.9
	Acne	46	10.5
	Other	4	0.9

Cosmetic procedures and attitudes

Out of the 439 participants, 103 (23.5%) reported having undergone cosmetic procedures, while 336 (76.5%) had not. Satisfaction with these procedures was varied: 12 participants (2.7%) were dissatisfied, 33 (7.5%) were neutral, and 52 (11.8%) were satisfied. Future intentions to undergo cosmetic procedures were expressed by 62 participants (14.1%), whereas 35 (8.0%) indicated no intention to do so. Complications related to procedures were reported by 13 participants (3.0%), with the majority (84, 19.1%) experiencing no complications (Table 3).

**Table 3:** Cosmetic procedures and attitudes (N=439) Data are represented as frequencies and percentages for cosmetic procedures and related attitudes among the study participants.

Variable		Frequency	Percent
Procedure Experience	No	336	76.5
	Yes	103	23.5
Satisfaction with Procedure	Dissatisfied	12	2.7
	Neutral	33	7.5
	Satisfied	52	11.8
Future Intentions	No	35	8.0
	Yes	62	14.1
Complications	No	84	19.1
	Yes	13	3.0

Attitudes towards cosmetic procedures

Participants had diverse attitudes towards cosmetic procedures. A majority of 221 participants (50.5%) agreed that cosmetic procedures create unrealistic beauty standards. Additionally, 256 participants (58.3%) viewed these procedures as a sign of affluence. Fewer participants believed that cosmetic procedures reflect vanity (104, 23.7%) or perceive a higher incidence of psychological issues associated with them (218, 49.7%) (Table 4).

**Table 4:** Attitudes towards cosmetic procedures (N=439) Data are represented as frequencies and percentages for attitudes towards cosmetic procedures among the study participants.

Perceptions of Cosmetic Procedures	Frequency	Percent
Agree that they create unrealistic beauty standards	221	50.5
Believe they reflect vanity	104	23.7
Perceive a higher incidence of psychological issues	218	49.7
View them as a sign of affluence	256	58.3

## Discussion

Our study reveals that a significant proportion of participants in Jazan, Saudi Arabia, have undergone cosmetic dermatological procedures, with 23.5% of respondents reporting previous experience with such treatments. This finding aligns with global trends where cosmetic procedures have become increasingly popular. However, the relatively high prevalence in Jazan highlights the region’s growing engagement with aesthetic enhancements. The factors driving this trend likely include societal pressures, media influence, and increased access to cosmetic treatments.

The demographic analysis indicates that cosmetic procedures are more common among younger individuals, with the majority of participants aged between 18 and 30 years. This trend mirrors global observations that younger populations are more likely to seek cosmetic enhancements, driven by social media influences and evolving beauty standards. Additionally, the predominance of higher education levels among those who have undergone procedures suggests that educational attainment may correlate with increased awareness and access to cosmetic dermatological options.

The study also explored psychological and dermatological conditions among participants. A notable proportion reported no previous psychological diagnoses (92.3%) and chronic skin conditions (76.5%). This suggests that individuals undergoing cosmetic procedures in Jazan are not primarily driven by psychological distress or chronic dermatological issues. Instead, the motivations may be more aligned with aesthetic preferences and social influences [5-10].

Satisfaction with cosmetic procedures varied, with a notable number of participants expressing dissatisfaction (2.7%) or neutrality (7.5%). The majority of those satisfied with the procedures may reflect the overall positive reception of cosmetic treatments. However, the future intentions of 14.1% of participants to undergo additional procedures indicate a continued interest in aesthetic enhancements, which could be influenced by ongoing trends and societal norms [8-15].

Attitudes towards cosmetic procedures revealed mixed perceptions. While 50.5% of participants agreed that cosmetic procedures create unrealistic beauty standards, 58.3% viewed them as a sign of affluence. These attitudes underscore the complex social dynamics surrounding cosmetic procedures, where they are simultaneously seen as symbols of both beauty and wealth. The perception of cosmetic procedures reflecting vanity (23.7%) and associated psychological issues (49.7%) further illustrates the nuanced views held by individuals regarding these treatments [11-13].

Limitations and future research

This study is not without limitations. The use of a non-probability sampling method may affect the generalizability of the findings beyond Jazan. Additionally, self-reported data may be subject to response bias. Future research should consider longitudinal studies to examine changes in cosmetic procedure trends over time and explore qualitative aspects of participants’ motivations and experiences in greater depth.

## Conclusions

In summary, this study provides a comprehensive overview of the prevalence and patterns of cosmetic dermatological procedures in Jazan, Saudi Arabia. Our findings indicate a significant engagement with cosmetic treatments, particularly among younger, well-educated individuals. Despite a generally positive reception, the study also reveals diverse attitudes towards cosmetic procedures, including concerns about unrealistic beauty standards and perceptions of vanity. These insights contribute to a deeper understanding of regional trends and highlight the need for continued research to explore the socio-cultural and psychological factors influencing cosmetic procedure use. The results underscore the importance of tailoring public health strategies and informing healthcare providers about the evolving landscape of cosmetic dermatology in Saudi Arabia.

## Appendices

Survey Questionnaire

Demographics

1. Age: 18-30 / 31-45 / 46-59 / 60+

2. Gender: / Male / Female / Other

3. Education: / High School or below / Diploma / Bachelor’s / Master’s or higher

4. Employment: / Employed / Unemployed / Retired

Cosmetic Procedures

5. Had cosmetic procedures? / Yes / No

6. If yes, which? / Botox / Fillers / Peels / Other: __________

7. Satisfaction: / Very satisfied / Satisfied / Neutral / Dissatisfied / Very dissatisfied

8. Future intentions: / Yes / No

Attitudes

9. Create unrealistic beauty standards? / Yes / No

10. Sign of affluence? / Yes / No

11. Reflect vanity? / Yes / No

12. Psychological concerns? / Yes / No
